# Pseudo-folliculite de la barbe chez les élèves policiers à Dakar : aspects épidémio-cliniques et facteurs de risque associés

**DOI:** 10.48327/mtsi.v4i2.2024.400

**Published:** 2024-04-18

**Authors:** Birame SECK, Moussa DIALLO, Mame Tene NDIAYE, Baha BOUIDIDA, Boubacar Ahy DIATTA, Maodo NDIAYE, Assane DIOP, Saer DIADIE, Ndèye Bougoul SECK, Fatimata LY, Suzanne Oumou NIANG

**Affiliations:** 1 Institut d’hygiène sociale, MHH3+7P4, Dakar, Sénégal; 2 Université Gaston Berger, BP 234, Saint-Louis, Sénégal; 3 Centre hospitalier régional de Saint Louis, Boulevard Abdoulaye Mar Diop, Saint Louis, Sénégal; 4 Hôpital Aristide Le Dantec, MH57+688, Rue Place 79, Dakar, Sénégal

**Keywords:** Pseudo-folliculite, Barbe, Aisselles, Pubis, Rasage, Élève policier, Homme, Femme, Noir africain, Dakar, Sénégal, Afrique subsaharienne, Pseudofolliculitis, Barbae, Shaving, Police students, Man, Woman, Black African, Dakar, Senegal, Sub-Saharan Africa

## Abstract

**Introduction:**

La pseudo-folliculite de la barbe (PFB) est une dermatose inflammatoire chronique, favorisée par le rasage et affectant essentiellement la barbe, mais aussi les autres zones du corps rasées (pubis, aisselles). Elle est particulièrement fréquente chez les Noirs africains, causant un important préjudice esthétique et professionnel. Cependant, il existe très peu de données disponibles pour cette affection, surtout en Afrique subsaharienne. Notre objectif était de déterminer les aspects épidémiologiques et cliniques, ainsi que les facteurs de risque associés à la survenue de la PFB à Dakar.

**Population et méthode:**

Il s’agit d’une étude transversale descriptive à visée analytique, réalisée en mars 2019, portant sur 655 élèves policiers pensionnaires de l’école nationale de police de Dakar, tous d’ascendance africaine et porteurs de cheveux crépus soumis à une obligation de rasage hebdomadaire. Le diagnostic de PFB était clinique.

**Résultats:**

Sur les 655 élèves policiers, 254 présentaient une PFB, soit une prévalence de 38,8 %. La prévalence de la PFB était de 43,7 % chez les hommes et de 11,9 % chez les femmes. L’âge moyen des élèves présentant la PFB était de 26,8 ans. L’âge de début de la PFB était compris entre 18 et 22 ans chez la majorité (53,9 %). Les lésions de PFB étaient prurigineuses dans 84,6 % des cas, à type de papules dans 96,8 % des cas et/ou de pustules dans 60,2 % des cas. La région sous-mandibulaire était le site le plus affecté (69,8 %). Des complications étaient notées dans 90,1 % des cas, surtout à type d’hyperpigmentation post-inflammatoire (87 %). Les facteurs de risque associés à la PFB étaient le sexe masculin, l’existence d’un antécédent familial de PFB, la peau à tendance chéloïdienne, l’association à une folliculite fibrosante de la nuque, l’utilisation de rasoirs à lame unique ou à tête fixe, le rasage à contre-sens du poil, le non-usage de produits de rasage et l’épilation à la cire.

**Conclusion:**

Notre étude confirme la fréquence élevée de la PFB dans cette population d’hommes noirs d’ascendance africaine. Une anomalie génétique révélée par le rasage doit être évoquée dans la survenue de la PFB, nécessitant ainsi d’autres études sur le plan génétique et immunohistochimique.

## Abstract (en)

**Background:**

Pseudofolliculitis barbae (PFB) is a chronic inflammatory skin disease favoured by shaving. It is particularly common among black Africans belonging to certain socio-professional categories who are obliged to shave. Its aesthetic and professional damage is very significant. However, very few data are available for this condition, especially in Sub-Saharan Africa.

**Objective:**

To determine the epidemiological and clinical aspects, and the risk factors associated with the occurrence of PFB in Dakar.

**Population and methods:**

This was a descriptive cross-sectional study conducted in March 2019, including 655 police students at the National Police Academy in Dakar, all of African descent and with curly hair, who were required to shave weekly and agreed to participate in this study. The diagnosis of PFB was clinically based. Data analysis was processed using Epi-info version 6.0 software. Pearson’s chi-square test was used for bivariate analysis with a significance threshold of p < 0.05. The Odds Ratio, with its 95% confidence interval, was used to determine the risk factors.

**Results:**

Among the 655 officers, 254 had PFB, with a prevalence of 38.8%. The prevalence of PFB was 43.7% in men (242 men out of 554) and 11.9% in women (12 women out of 101). The average age of patients with PFB was 26.80 years (± 2.59), ranging from 22 to 36 years. The age of onset of PFB was between 18 and 20 years for the majority (39.8%), with a mean age of onset of 22.2 years (± 3.6). PFB lesions were pruritic in 84.6% of cases, papular in 96.8%, and/or pustular in 60.2%. The submandibular region was the most affected site (69.8%). Complications were noted in 90.1% of cases in the form of post-inflammatory hyperpigmentation (87%) and keloid scars (3.1%). The risk factors associated with PFB were male sex (p<0.0001; OR=5.7; CI95% [3.07-10.75]), family history of PFB (p<0.0001; OR=5; CI95% [3.35-7.37]), keloid-prone skin (p<0.0001; OR=2.9; CI95% [1.63-4.96]), association with acne keloidalis nuchae (p<0.0001; OR=8.8; CI95% [5.55-14.08]), use of a single-blade razor (p<0.0001; OR=2.5; CI95% [1.69-3.70]), use of a fixed-head razor (p<0.0001; OR=1.8 CI95% [1.28-2.77]), shaving against the grain (p<0.0001; OR = 6.3; CI95%= [4.33-9.08]), non-use of shaving products (p = 0.009; OR = 1.5; CI95%= [1.06-2]) and waxing (p<0.004; OR=2.7; CI95% [1.33-5.77]). On the other hand, the use of clippers (p<0.0001; OR = 0.5 CI95% [0.33-0.65]), pre-shave products (p<0.0001; OR = 0.4 CI95% [0.29-0.61]) and the use of razors with movable heads (p<0.0009; OR = 0.2 CI95% [0.17-0.35]) were protective factors against PFB.

**Conclusion:**

Our study confirms the high incidence of PFB in this population of black men of African descent. A genetic abnormality revealed by shaving must be evoked in the occurrence of PFB. Further genetic and immunohistochemical studies would be needed to support this hypothesis.

## Introduction

La pseudo-folliculite de la barbe (PFB) est une dermatose inflammatoire chronique, favorisée par le rasage, affectant principalement les hommes jeunes d’ascendance africaine aux cheveux fortement convolutés dits « crépus » [9]. Sa prévalence serait comprise entre 45 % et 83 %, selon les différentes études menées au sein de la population afro-américaine [1, 13, 15]. Cette affection peut également se rencontrer chez les femmes se rasant le visage, les aisselles et le pubis [13]. La PFB est une affection affichante et potentiellement défigurante, responsable d’un préjudice esthétique majeur. Par ailleurs, elle peut poser un sérieux problème sur le plan professionnel, notamment dans les corps militaires et paramilitaires, soumis aux obligations de rasage [1, 15].

Malgré sa fréquence et son important retentissement psychologique et professionnel, très peu de travaux ont été consacrés à cette affection. À notre connaissance, aucune étude sur la PFB n’a encore été réalisée en Afrique subsaharienne. Par ailleurs, l’absence de preuves robustes sur les facteurs de risque de cette affection explique le manque de consensus sur les recommandations de prévention et de traitement de la PFB. Ainsi, jusqu’à ce jour, la prévention et la prise en charge de cette affection reposent uniquement sur des avis empiriques d’experts individuels.

L’objectif de notre étude était de déterminer les aspects épidémiologiques, cliniques, ainsi que les facteurs associés à la survenue de la PFB à Dakar.

## Population-et méthodes

Il s’agit d’une étude transversale descriptive à visée analytique, menée à l’école nationale de police de Dakar en mars 2019, portant sur 655 élèves policiers consentants, tous d’ascendance africaine et porteurs de cheveux crépus, soumis aux obligations de rasage hebdomadaire. Le diagnostic clinique de PFB a été retenu devant la présence de papules inflammatoires et/ou de pustules, récidivantes siégeant sur les zones soumises au rasage.

Une folliculite fibrosante de la nuque a été systématiquement recherchée. Son diagnostic repose sur la présence de papules et de plaques fibreuses alopéciantes confinées à la nuque. Les données ont été collectées grâce à une fiche d’enquête. Un exposé sur la PFB comportant une iconographie a été au préalable présenté aux participants à l’issue duquel chaque élève policier a répondu librement aux items de la fiche d’enquête. Par la suite, les élèves présentant une PFB ont été examinés par deux dermatologues, afin de préciser la nature des lésions et leur topographie. Un traitement et des conseils adaptés ont été ensuite fournis aux élèves présentant une PFB.

Les données recueillies ont été exploitées grâce au logiciel Epi-info version 6.0. Le test du Khi2 de Pearson a été utilisé pour l’analyse bivariée avec un seuil de significativité pour p < 0,05. Des Odds-ratio, encadrés par leur intervalle de confiance à 95 %, ont été calculés afin de déterminer les facteurs de risque associés à la survenue de PFB.

## Résultats

Les 655 élèves policiers inclus dans l’étude ont été répartis en 554 hommes et 101 femmes. Parmi eux, 254 avaient une PFB, soit une prévalence de 38,8 %. Les principaux diagnostics différentiels ont été l’acné vulgaire et le sycosis de la barbe. La prévalence de la PFB était de 43.7 % chez les hommes (soit 242 hommes sur 554) contre 11,9 % chez les femmes (soit 12 femmes sur 101), avec une différence statistiquement significative (p<0,001). L’âge moyen des sujets présentant une PFB était de 26.8 (± 2,6) ans avec un âge médian de 26 ans. Chez la majorité des élèves (53,9 %), l’âge de début de la PFB était compris entre 18 et 22 ans, avec un âge moyen de début de 22,2 (± 3,6) ans. Des antécédents familiaux de PFB ont été rapportés dans 39,8 % des cas. La PFB était associée à une folliculite fibrosante de la nuque dans 38,6 % des cas.

**Figure 1:**
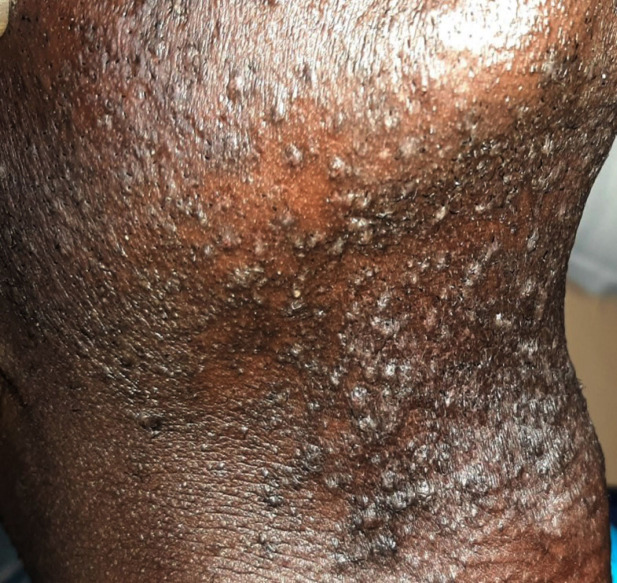
Papules et pustules au cours de la PFB Papules and pustules in PFB

Les lésions de PFB étaient prurigineuses dans 84,6 % des cas et douloureuses dans 66,1 %. Elles étaient à type de papules dans 96,8 % des cas et de pustules dans 60,2 % (Fig. 1). Des signes d’incarnation de poil recourbé ont été observés dans 27,9 % des cas. Chez les hommes, les sites les plus affectés étaient la région sousmandibulaire (69,8 %), la région mandibulaire (52,1 %) et la face antérieure du cou (46,3 %). Chez les femmes, les lésions étaient le plus souvent localisées au pubis (66,7 %) et aux creux axillaires (25 %). Les différentes localisations selon le sexe sont détaillées dans le Tableau I. Des complications ont été notées dans 90,1 % des cas, à type d’hyperpigmentation post-inflammatoire dans 87 % des cas et de cicatrices chéloïdiennes dans 3,1 %.

**Tableau I:** Topographie des lésions de PFB selon le sexe Topography of PFB lesions by sex

Topographie	Hommes n (%)	Femmes n (%)
Région sous-mandibulaire	177 (73,1)	0
Région mandibulaire	126 (52,1)	0
Face antérieure du cou	112 (46,3)	1 (8,3)
Menton	93 (38,4)	1 (8,3)
Joues	48 (19,8)	0
Pubis	36 (14,9)	8 (66,7)
Creux axillaire	25 (10,3)	3 (25)
Jambe	5 (2,1)	2 (16,7)

Concernant les méthodes d’épilation, tous les hommes ayant une PFB se rasaient régulièrement avec des rasoirs mécaniques à lame, contre 66,7 % chez les femmes ayant une PFB (Tableau II).

**Tableau II:** Pratiques d’épilation chez les hommes et les femmes atteints de PFB Hair removal practices in men and women with PFB

Pratiques	Hommes n (%)	Femmes n (%)
Rasage à la lame	242 (100)	8 (66,7)
Épilation à la tondeuse électrique	77 (31,8)	6 (50)
Épilation à la cire	18 (7,4)	2 (16,7)
Épilation à la pince	6 (2,5)	3 (25)
Rasage à contre sens	127 (52,5)	5 (41,6)
Produits prérasage	45 (18,6)	4 (33,3)
Produits post-rasage	55 (22,7)	5 (41,6)

Les traitements fournis étaient essentiellement à base de trétinoïne (48 % des cas), de doxycycline (40,5 %) et de dermocorticoïdes (15,7 %). Après un recul de 3 mois, l’évolution était marquée par une amélioration dans 72,4 % des cas, un état stationnaire dans 25,2 % et une aggravation dans 4,3 % des cas.

Les facteurs de risque associés à la PFB (Tableau III) étaient : le sexe masculin, l’existence d’un antécédent familial de PFB, les antécédents de chéloïde, l’association à une folliculite fibrosante de la nuque, l’utilisation de rasoirs mécaniques à lame unique, à tête fixe, le rasage à contre-sens du poil, le non-usage de produits de rasage et l’épilation à la cire. Par contre, la taille à la tondeuse, l’utilisation de produits de prérasage et de produits post-rasage, et l’utilisation de rasoirs à tête mobile étaient des facteurs protecteurs contre la PFB. Les produits de prérasage étaient essentiellement à base de savon (n=27) ou de mousse (n=12) et les produits post-rasage étaient essentiellement à base d’alcool (n=31) ou de beurre de karité (n=23).

**Tableau III:** Facteurs de risques associés à la PFB, analyse bivariée Risk factors associated to PFB, bivariate analysis

Variables	PFB		OR	IC à 95 %	P
	Présente (n)	Absente (n)			
**Sexe**					
**masculin**	242	312	5,7	3,07-10,75	< 0,0001
**féminin**	12	89			
**Antécédent familial de PFB**					
**oui**	101	153	5	3,35-7,37	< 0,0001
**non**	47	354			
**Antécédent de chéloïde**					
**oui**	36	22	2,9	1,63-4,96	< 0,0001
**non**	218	379			
**Folliculite fibrosante de la nuque**					
**oui**	99	27	8,8	5,55-14,08	< 0,0001
**non**	155	374			
**Types d’épilation**					
**à la cire**	20	12	2,7	1,33-5,77	0,004
**à la tondeuse**	83	204	0,5	0,33-0,65	< 0,0001
**à la pince**	9	12	-	-	0,34
**Rasage à contre sens du poil**					
**oui**	132	59	6,3	4,33-9,08	< 0,0001
**non**	122	342			
**Utilisation de produits**					
**avant le rasage**	49	144	0,4	0,29-0,61	< 0,0001
**après le rasage**	60	66	-	-	0,01
**aucun**	145	191	1,5	1,06-2	0,009
**Type de rasoir mécanique**					
**à lame unique**	212	268	2,5	1,69-3,70	< 0,0001
**à lame multiple**	120	191	-	-	0,46
**à tête fixe**	208	283	1,8	1,28-2,77	< 0,0001
**à tête mobile**	51	203	0,2	0,17-0,35	< 0,0001

## Discussion

À notre connaissance, il s’agit de la première étude sur la PFB réalisée en Afrique subsaharienne. Le site de recrutement choisi constitue un observatoire privilégié de la PFB du fait des obligations de rasages hebdomadaires auxquelles sont soumis les pensionnaires de l’école nationale de police. Nous avons contacté un des responsables de formation dans l’école de police pour encore plus de détails. Il nous a confirmé les obligations de rasage pour tous les élèves policiers sans distinction de sexe. Ces élèves policiers subissent, en effet, une formation militaire selon presque les mêmes conditions que dans l’armée sénégalaise. Et pour les femmes, il existe des instructrices chargées de veiller au strict respect de ces dispositions.

Notre étude apporte des données nouvelles sur les facteurs de risque associés à la survenue de la PFB.

Il ressort de notre étude que la PFB est une affection très fréquente chez les pensionnaires de l’école nationale de police de Dakar avec une prévalence de 38,8 %. Cette observation pourrait être étendue aux autres corps militaires et paramilitaires du Sénégal qui sont soumis aux mêmes obligations de rasage que les pensionnaires de l’école nationale de police.

Cependant, des prévalences plus importantes ont été rapportées aux États-Unis, au sein de la population militaire afro-américaine, avec des fréquences allant de 45 à 83 % [1, 13, 15]. La fréquence de la PFB chez les sujets d’ascendance africaine s’expliquerait avant tout par la forme fortement convolutée dite « crépue » de leur pilosité, avec une section de la tige pilaire aplatie. Les follicules pileux chez les sujets d’ascendance africaine sont incurvés et implantés très obliquement dans la peau, avec une concavité dirigée vers l’épiderme, ce qui favorise une incarnation du poil lors du rasage, induisant ainsi une réponse inflammatoire granulomateuse dès que le poil repousse dans le derme [13, 16]. Dans notre étude, les hommes étaient plus affectés que les femmes. La prévalence de la PFB était de 43,7 % chez les hommes contre 11,9 % chez les femmes avec une différence statistiquement significative. Les différences des pratiques d’épilation entre les hommes et les femmes et la texture de la pilosité des hommes noirs plus crépue et plus rugueuse pourraient expliquer ces résultats.

La génétique semble également jouer un rôle important, expliquant dans notre étude l’association significative entre l’existence d’un antécédent familial de PFB et la survenue de l’affection. Winter *et al.* ont démontré que le déterminant génétique de la PFB est lié au polymorphisme Ala12Thr situé dans le gène *K6hf* (hair follicle companion layer-specific keratin 6) de la kératine [17]. Ils ont rapporté que 36 % des hommes se rasant régulièrement et porteurs de ce polymorphisme développent une PFB contre 9 % chez les témoins avec un risque relatif de 6,12 [17]. De plus, ils ont précisé que la mutation Ala12Thr du gène K6hf était plus fréquente chez les Afro-américains que chez les autres. En effet, sur les 90 Afro-américains inclus dans cette étude, 33 présentaient cette mutation (36,7 %), tandis que chez les 110 autres Américains, seuls 12 avaient ladite mutation (10,9 %).

Chez la plupart des élèves policiers, la PFB débute à un âge jeune, conformément aux données de la littérature. En effet, selon plusieurs études, la PFB débute après la puberté avec l’apparition de la pilosité sexuelle secondaire qui pousse de manière significative, avec des poils devenant épais et bouclés, ce qui nécessite souvent un rasage régulier [6, 12, 13]. L’aspect clinique de la PFB était caractéristique chez la quasi-totalité des participants. On observait en effet des papules inflammatoires prurigineuses, souvent associées à des pustules, au sommet desquelles apparaissait une incarnation pilaire qui était bien visible chez près du tiers des cas. Les lésions de PFB prédominaient chez les hommes au niveau des régions sous-mandibulaire et mandibulaire, au cou et au menton. La prédominance de ces localisations a été également rapportée dans d’autres études réalisées chez les Noirs afro-américains [1, 13]. L’atteinte élective de ces zones pourrait s’expliquer, d’une part par l’irrégularité du relief de la peau au niveau de ces régions, constituant un véritable obstacle au rasage complet, et d’autre part par la densité accrue des follicules pileux dans ces régions [5, 10]. Ces zones sont également soumises à une fréquence accrue du rasage chez les hommes. Par contre, chez les femmes, la topographie élective des lésions de PFB était plutôt le pubis, suivie de la région axillaire.

Un seul cas d’atteinte du cou et du menton a été noté dans notre étude chez une femme qui présentait également un hirsutisme et qui se rasait régulièrement la barbe.

L’existence d’une peau susceptible de faire des chéloïdes, en particulier objectivée par un antécédent de chéloïde, a été significativement associée au risque de survenue de la PFB dans notre étude. À notre connaissance, cette association n’a pas encore été rapportée dans la littérature. Néanmoins, il a été rapporté une incidence significativement plus élevée de la susceptibilité à faire des cicatrices chéloïdes chez les sujets à peau fortement pigmentée par rapport aux autres phototypes, au même titre que la PFB [2]. Ceci nous amène à penser que la PFB et les chéloïdes pourraient partager en partie les mêmes prédispositions génétiques. Des études génétiques seront nécessaires pour pouvoir étayer cette hypothèse.

Nos résultats ont également montré une association significative entre la folliculite fibrosante de la nuque et la PFB. East-Innis *et al.* ont rapporté un résultat similaire chez les Afro-Caribéens [4]. La folliculite fibrosante de la nuque, encore appelée acné chéloïdienne de la nuque, est une forme chronique de folliculite cicatricielle observée principalement chez les hommes d’ascendance africaine ayant des cheveux crépus [11]. Cette affection partagerait probablement avec la PFB les mêmes mécanismes physiopathologiques d’incarnation pilaire avec une forte implication génétique [8]. En plus, ces deux affections sont soumises aux mêmes conditions de rasage et aux mêmes obligations dans les corps militaires et paramilitaires.

Dans notre étude, différentes méthodes de rasage ont été associées au risque de survenue de la PFB. Il s’agissait de l’utilisation de rasoirs à lame unique ou à tête fixe, du rasage à contre-sens du poil et de l’épilation à la cire. Il est important de préciser que les rasoirs utilisés par nos participants étaient tous à usage individuel. Un des responsables de l’école nous a précisé qu’un rasoir pouvait servir deux à trois fois chez un même individu, mais qu’il était formellement interdit de s’échanger un rasoir entre camarade pour « éviter tout risque de transmission de maladie ». Les études sur l’influence des techniques de rasage dans la PFB sont rares et très controversées. Il a été rapporté que l’utilisation de rasoir à lame multiple favorisait la pénétration transfolliculaire des poils en croissance augmentant ainsi le risque de PFB, amenant les auteurs à recommander l’utilisation de rasoir à lame unique, ce qui est contraire à nos résultats [7]. Des études plus récentes ont suggéré que le nombre de lame n’influençait pas le risque de PFB, mais ce sont plutôt les mauvaises techniques de rasage, telles que le rasage à contre-sens du poil, le rasage trop court des poils avec un rasoir à lame, l’étirement du poil pendant le rasage et le rasage à sec, qui prédisposaient au risque de PFB [3, 6]. Concernant le rasage à contre-sens du poil, beaucoup d’auteurs se sont accordés sur le fait qu’il favorise l’incarnation pilaire et donc la PFB [14]. Il devrait ainsi être évité dans les mesures préventives. Par ailleurs, notre étude a révélé que certains facteurs pourraient, à l’inverse, réduire le risque de PFB. Il s’agissait de taille à la tondeuse électrique, de l’utilisation de produits de prérasage et post-rasage, et de l’utilisation de rasoir à tête mobile. La plupart des études sur les techniques de rasage recommandent l’usage de la tondeuse électrique et l’hydratation avant et après rasage [6, 12]. Les tondeuses électriques sont en effet équipées d’une grille de protection dont la largeur est réglable, ce qui permet de laisser un minimum de 1 mm de poil nécessaire pour éviter une pénétration transfolliculaire de la tige pilaire. En ce qui concerne les produits pré et post-rasage, leur utilisation améliore l’hydratation des poils de la barbe, les rendant plus doux et plus faciles à couper, et désinfecte également la surface cutanée avec l’usage du savon et de l’alcool, comme c’était le cas chez nos participants.

## Conclusion

La PFB est une pathologie d’incarnation pilaire très fréquente chez les hommes noirs d’ascendance africaine, souvent d’âge jeune, porteurs de cheveux crépus et se rasant régulièrement la zone de la barbe. Cette affection semble survenir chez des sujets génétiquement prédisposés, favorisée par certaines méthodes de rasage inadaptées et l’absence de soins adéquats avant et après rasage. Des moyens efficaces de prévention existent pour cette affection. Des études sur le plan génétique et immunohistochimique seront nécessaires pour mieux caractériser la PFB.

## Contribution des auteurs

Birame SECK : conception de l’étude, investigateur principal, rédaction du protocole et du manuscrit

Moussa DIALLO : supervision de l’étude, correction du protocole et du manuscrit Mame Tene NDIAYE : participation à la collecte des données et à l’analyse statistique Baha BOUIDIDA : participation à la collecte des données

Boubacar Ahy DIATTA : validation du manuscrit

Maodo NDIAYE : validation du manuscrit

Assane DIOP : validation du manuscrit

Saer DIADIE : validation du manuscrit

Ndeye Bougoul SECK : validation du manuscrit

Fatimata LY : validation du manuscrit

Suzanne Oumou NIANG : relecture, validation du protocole et du manuscrit

## Liens d’intérêts

Les auteurs déclarent ne pas avoir de liens d’intérêts.
